# Supramolecular Solvent-Based Extraction of Bisphenols and Alkylphenols in Botanical Dietary Supplements Prior to HPLC–MS/MS Analysis

**DOI:** 10.3390/foods14213768

**Published:** 2025-11-03

**Authors:** Yalei Dong, Huijun Liu, Yasen Qiao, Haiyan Wang

**Affiliations:** 1 National Institute for Food and Drug Control, Beijing 100050, China; dongyalei@nifdc.org.cn; 2 Department of Food Science and Engineering, Beijing University of Agriculture, Beijing 102206, China; huijunliu78@163.com

**Keywords:** supramolecular solvent, pretreatment, bisphenols, alkylphenols, botanical dietary supplement, HPLC-MS/MS

## Abstract

Dietary supplements provide essential nutrients and bioactive compounds that enhance health and traditional therapies. However, the quality and composition of these supplements can vary significantly, potentially containing inconsistent levels of active ingredients or undisclosed risk substances. Due to the current extensive industrial applications, bisphenols (BPs) and alkylphenols (APs) have become environmentally ubiquitous. Substantial evidence indicates that these compounds exhibit endocrine-disrupting properties, posing potential health risks to humans. The detection of trace-level BPs and APs in dietary supplements is critical. This study developed a supramolecular solvent (SUPRAS) from a water/THF/1-hexanol system under mild conditions for analyzing 19 BPs and APs in commercial botanical dietary supplements. After optimizing SUPRAS preparation and extraction parameters, we established a SUPRAS–HPLC–MS/MS method enabling one-step extraction/cleanup within 10 min for tablets, capsules, and oral liquids, with high sensitivity and simplicity. The method scored 0.71 (out of 1) on the AGREE metric, confirming its green profile. Detectable levels of bisphenol A (178.7–452.6 μg/kg) and 4-pentylphenol (145.3 μg/kg) in marketed products highlight potential health risks from botanical dietary supplement-derived exposure.

## 1. Introduction

Dietary supplements play a vital role in bridging nutritional gaps and supporting overall health, particularly for individuals with inadequate dietary intake or specific health conditions [1]. Emerging evidence highlights their potential in disease prevention and health promotion [2,3]. For instance, medium-chain triglycerides have been shown to improve glucose metabolism and muscle density in elderly populations [4]. Zinc and folic acid supplements have been associated with enhanced sperm quality in men with infertility [5,6,7]. However, safety concerns regarding dietary supplements also remain a critical issue. Regulatory shortcomings permit insufficient premarket safety assessments, enabling the presence of undeclared ingredients [8], residual heavy metals [9], or risky contaminants [10] in dietary supplements. To safeguard public health, particularly among vulnerable populations, the identification of hazardous substances in dietary supplements is essential for a comprehensive risk assessment.

Bisphenols (BPs) [11] are primarily released from plastic products and food packaging, while alkylphenols (APs) [12] such as nonylphenol and octylphenol predominantly originate from detergents, pesticides, and plastic additives, and so on. These contaminants permeate into food and water systems [13,14] through industrial processes, packaging material migration, or contaminated agricultural irrigation. Extensive research has established both BPs and APs as potent endocrine-disrupting chemicals (EDCs) associated with diverse adverse health effects. Exposure to these compounds was reported to link to reproductive disorders, metabolic diseases (e.g., diabetes), and cancer [15,16,17,18]. Dietary supplements are vulnerable to contamination during production or packaging as evidenced by detecting BPA in sports supplements (0.852–2.892 ng/mg) [19]. APs may infiltrate plant-derived supplements via bioaccumulation in crops or leaking from plastic packaging materials. Consequently, comprehensive profiling of the occurrence and concentration ranges of various BP and AP analogs in botanical dietary supplements represents could be a valuable research priority.

Current analytical methods for monitoring trace-level contaminants in food stuff primarily employ GC–MS and LC–MS techniques. Given their low volatility and wide polarity range, LC–MS demonstrates superior analytical performance for BP and AP analogs compared to alternative techniques [20]. Nevertheless, the diverse formulations and complex matrices of botanical dietary supplements necessitate efficient sample pretreatment protocols prior to instrumental detection. Existing techniques included solid-phase microextraction (SPME) [21], magnetic solid-phase extraction (MSPE) [22], dispersive liquid–liquid microextraction (DLLME) [23], and conventional solid-phase extraction (SPE) [24]. However, SPME suffers from fiber durability issues, MSPE requires complex nanomaterial synthesis, DLLME faces reproducibility challenges, and SPE consumes large volumes of toxic solvents. These limitations collectively encouraged the development of novel pretreatment methods that simultaneously achieve environmental compatibility and high processing efficiency.

Supramolecular solvents (SUPRAS) are nanostructured liquids formed through the self-assembly and coacervation of amphiphilic molecules [25]. Their unique microenvironments enable multi-mechanism extraction (hydrogen bonding, ionic, and hydrophobic interactions) with minimal organic solvent consumption [26]. SUPRAS-based extraction technology possesses key advantages, including high enrichment efficiency, rapid mass transfer, and tunable properties through pH/temperature modulation. These combined properties facilitate the development of a universal sample pretreatment methodology for trace analytes determination across varied matrices, such as indoor dust [27,28], thermal paper [29], sediment [30], hair, and wastewater [31]. Furthermore, SUPRAS was evaluated alongside QuEChERS for 14 bisphenols in bee pollen via UHPLC–MS/MS [32], proving better solvent efficiency. SUPRAS have demonstrated versatile applications for trace contaminant analysis across diverse food matrices, including bisphenols and analogs in food packaging materials, benzophenones in orange juice [33], 12 regulated mycotoxins in five food groups [34], and bioactive compounds in fruit [35], offering rapid, eco-friendly extraction with superior matrix compatibility. Nevertheless, the utilization of SUPRAS for the extraction and determination of BP and AP contaminants in botanical dietary supplements has been rarely reported to date.

Herein, this study developed a novel SUPRAS-assisted extraction method coupled with HPLC–MS/MS for the simultaneous determination of APs and BPs in botanical dietary supplements. Critical parameters affecting extraction efficiency were systematically optimized, and the LC–MS conditions were carefully refined. The validated analytical approach confirmed the presence of AP and BP contaminants in commercial botanical dietary supplements. To assess the health risks of the detected contaminants, a simplified protocol was employed based on the monitoring data, highlighting potential exposure risks to consumers.

## 2. Materials and Methods

### 2.1. Chemicals and Reagents

Bisphenol A (BPA, 99.8%), Bisphenol B (BPB, 99.5%), Tetrabromobisphenol A (TBBPA, 99.4%), Bisphenol BP (BPBP, 98.8%), and 4-Nonylphenol (4-NP, 99.7%) were purchased from Dr. Ehrenstorfer (Augsburg, Germany). Bisphenol AF (BPAF, 99.5%) and 4-Heptylphenol (4-HeptyP, 99.5%) came from ChemService (West Chester, PA, USA) and Tetrachlorobisphenol A (TCBPA, 98%) from TRC (Toronto, ON, Canada). Bisphenol C (BPC, 1000 μg/mL), Bisphenol G (BPG, 1001.6 μg/mL), Bisphenol P (BPP, 1000 μg/mL), Bisphenol Z (BPZ, 1000 μg/mL), Bisphenol AP (BPAP, 1005.0 μg/mL), 4-Butylphenol (4-BP, 1000 μg/mL), 4-tert-Butylphenol (4-t-BP, 1000 μg/mL), and 4-Hexylphenol (4-HexylP, 1000 μg/mL) solution in methanol were obtained from Alta scientific (Tianjin, China) as 1 mL ampoules. Bisphenol E (BPE, 100%), 4-Pentylphenol (4-PP, 99.3%), and 4-tert-Octylphenol (4-t-OP, 98.0%) were acquired as solids from BePure reference material procedure (Beijing, China). The basic information and physicochemical parameters of the 19 compounds are presented in Table S1.

Methanol, acetonitrile, and tetrahydrofuran (THF) were LC grade and supplied by Fisher Scientific (Waltham, MA, USA). Ammonium fluoride was LC–MS grade and came from Honeywell International Inc. (Morris Plains, NJ, USA). Alkyl alcohols including 1-pentanol (C5), 1-hexanol (C6), and 1-nonanol (C10) were analytical grade and came from Sinopharm Chemical Reagent Co., Ltd. (Shanghai, China). 1-heptanol (C7), 1-octanol (C8), and 1-nonanol (C9) were analytical grade and obtained from Tokyo Chemical Industry (Tokyo, Japan). Ultrapure water was used throughout.

### 2.2. Apparatus

The HPLC (AD, Shimadzu, Japan) coupled with a triple quadrupole system (Triple Quad 4500, AB SCIEX, Framingham, MA, USA) equipped with an electrospray ion (ESI) source was performed for HPLC–MS/MS analysis. Vortex mixer, centrifuge, and electronic balance (Mettler Toledo, Greifensee, Switzerland) were used.

### 2.3. Standard Solutions

The standard stock solutions (~1 mg/mL) of each analyte were obtained commercially or prepared by dissolving 10 mg of the solid reference substances in 10 mL of acetonitrile. Single-component stock solutions were stored at 2–8 °C. For the mixed standard intermediate solution (10 μg/mL), 100 μL of each stock solution was transferred to a 10 mL volumetric flask, diluted with methanol, and mixed thoroughly.

Approximately 0.1 g of blank samples (pre-analyzed capsules, tablets, and oral liquids matrices containing no detectable levels of BPs and APs) were weighed into 10 mL screw-cap centrifuge tubes. After adding 4 mL of SUPRAS, the mixture was vortexed for 1 min and centrifuged at 7012× *g* for 5 min. The supernatants were collected as matrix blank solutions. A series of matrix-matched calibration standards were prepared by diluting different volumes of the mixed standard intermediate solution with the matrix blanks.

### 2.4. Instrument Parameters

To minimize the background interference, a Shim-pack Scepter C_18_-120 (3.0 × 50 mm, 3 μm) column was used as pre-column. A poroshell 120 EC-C_18_ (2.7 μm, 2.1 × 100 mm) column was employed for separation. Gradient elution was performed with the mobile phase which consisted of 0.2 mmol/L ammonium fluoride aqueous solution (A) and methanol (B) at 0.3 mL/min with the following gradient: 0~1 min: 20% B; 1~3 min: 20%~60% B; 3~15 min: 60%~90% B; 15~18 min: 90% B; 18~18.1 min: 90%~20% B; 18.1~20 min: 20% B. The column temperature was maintained at 40 °C and the sample injection volume was 5 μL. The 19 compounds were analyzed using Scheduled MRM with negative ESI. MS parameters: IonSpray Voltage −4500 V, source temperature 450 °C, curtain gas 10 psi, GS1/GS2 55 psi. Compound-specific parameters (precursor/product ions, DP, CE) were optimized and listed in Table 1.

### 2.5. Preparation of SUPRAS

The preparation method of the SUPRAS was similar to that described in our previous research [36]. In a 50 mL Corning screw-cap centrifuge tube, 2 mL of 1-hexanol was firstly mixed with 8 mL of THF. Ultrapure water was then rapidly added to adjust the total volume to 40 mL. The mixture was vortexed for 1 min and centrifuged at 7012× *g* for 5 min, during which SUPRAS spontaneously formed and separated into the upper phase. Approximately 5 mL of the SUPRAS phase was transferred to an airtight brown glass bottle and stored at 4 °C for at least one month.

### 2.6. Sample Pretreatment with SUPRAS

Twenty-six product batches were acquired through randomized online sampling from the Chinese market. The products encompassed a range of brands and health benefit claims in major dosage forms, including tablets, capsules, and oral liquids, primarily packaged in plastic, aluminum–plastic, or glass bottles. All samples shared a common formulation basis of plant-based raw materials, particularly medicinal plants. Following assignment of unique codes, they were stored under cool, dry conditions until analysis. Prior to sample preparation, the tablets were ground into homogeneous powder, while capsule contents and oral solutions were directly extracted using SUPRAS. A 15 mL Corning screw-cap centrifuge tube was first charged with 0.1 g of sample. Then 4 mL of SUPRAS was added to the tube, and the mixture was agitated for 1 min. Then, the supernatant was obtained after centrifuging at 6533× *g* for 5 min. The extract was mixed with an equal volume of methanol prior to HPLC–MS/MS analysis.

### 2.7. Health Risk Assessment

A preliminary assessment was performed on the basis of limited sampling to reveal potential health risks associated with contaminated product. The estimated daily intake (EDI) of the detected BPA and 4-PP was determined by the Formula (1) [37]:

(1) EDI = (C × I)/BW

where C (ng/g) is the content of analytes in samples, I (g/day) is the daily consumption amount of dietary supplements, and BW (kg) is the average body weight, which is 60 kg for a Chinese adult. In 2023, EFSA considerably lowered the value of the tolerable daily intake (TDI) of BPA to be 0.2 ng/kg·BW/day [38] compared to the value issued in 2015 (4 µg/kg·BW/day). For health risk assessment, hazard quotient (HQ) [39], which was dimensionless, was calculated using the following Equation (2):

(2) HQ = EDI/TDI

The hazard index (HI), for evaluating the human health risk resulting from the exposure of multiple residues of bisphenol analog, was further calculated as the sum of HQ for bisphenol analogs using the Formula (3):

(3) HI = ΣHQ = HQ_BPA_ + HQ_4-PP_

### 2.8. Data Analysis

Microsoft Excel (2010) was employed to manage sample information and mass spectrometry data, perform statistical analysis, and conduct risk assessment calculations and tabulation. Origin software (9.0) facilitated data visualization and graphing. During the condition optimization, *p*-values were calculated by performing an analysis of variance (ANOVA) using R software (version 4.2.0, https://www.R-project.org/; The R Foundation, Vienna, Austria).

## 3. Results and Discussion

### 3.1. Control of Background Interference

Given the potential presence of BPs and APs as environmental contaminants, strict quality control measures were implemented to minimize background interference. Preliminary monitoring revealed detectable levels of BPs and APs in plastic droppers and filter membranes. Consequently, these consumables were systematically excluded from all experimental procedures. Meanwhile, to monitor potential contamination, blank solvents and samples were routinely analyzed throughout the injection sequence, with two blank injections systematically inserted after every ten samples to evaluate carryover. Any detected contamination triggered immediate troubleshooting and sample re-processing. The autosampler needle received rigorous washing with 50% methanol–water between injections to minimize cross-contamination.

### 3.2. Chromatographic Conditions Optimization

In chromatographic analysis, both peak morphology and signal response are critically influenced by the composition and concentration of mobile phase additives. For method optimization, a 50 μg/L mixed standard solution was employed to evaluate the mobile phase conditions. The target compounds, containing phenolic hydroxyl groups, are prone to deprotonation in negative ionization mode, predominantly forming [M-H]^−^ ions. As alkaline conditions theoretically promote ionization efficiency, we systematically evaluated four additives, including 0.1% (*v*/*v*) aqueous ammonia, 5 mmol/L ammonium formate, 5 mmol/L ammonium acetate, and 0.05 mmol/L ammonium fluoride. Results demonstrated that the first three additives showed no significant improvement in the ionization efficiency of target compounds. Notably, ammonia introduction increased background noise, compromising low-concentration detection. In contrast, low-concentration ammonium fluoride markedly enhanced the target response. The reason is likely the strong proton affinity of fluoride ions: these ions competitively capture protons, thereby improving the ionization of the compound in the negative mode [40]. Subsequently, the concentration of ammonium fluoride was systematically evaluated (0, 0.1, 0.2, 0.5, and 1 mmol/L). Signal responses for all target compounds increased with ammonium fluoride concentration up to 0.2 mmol/L, where most analytes reached maximum response intensity. The final mobile phase composition was therefore established as methanol and 0.2 mmol/L ammonium fluoride aqueous solution.

### 3.3. Optimization of Mass Spectrometry Conditions

Mass spectrometric parameters were optimized in MS-only mode through direct infusion of 100 μg/L standard solutions using a syringe pump. The optimization procedure consisted of three sequential steps: (1) precursor ion identification via Q1 Full Scan within the expected mass range of each analyte; (2) selection of the two most abundant product ions in Product Ion Scan mode, designated as quantitative and qualitative transitions, respectively; and (3) refinement of critical parameters including declustering potential (DP) and collision energy (CE) in multiple reaction monitoring (MRM) mode to achieve optimal sensitivity. For APs, which characteristically produced only one dominant product ion per precursor, a complementary transition was established using the isotopic peak as an alternative precursor. For example, 4-BP was monitored using two transitions, including 149.1/105.7 and 150.2/106.7, and the latter was qualitative transitions derived from the isotopic precursor.

**Figure 1:**
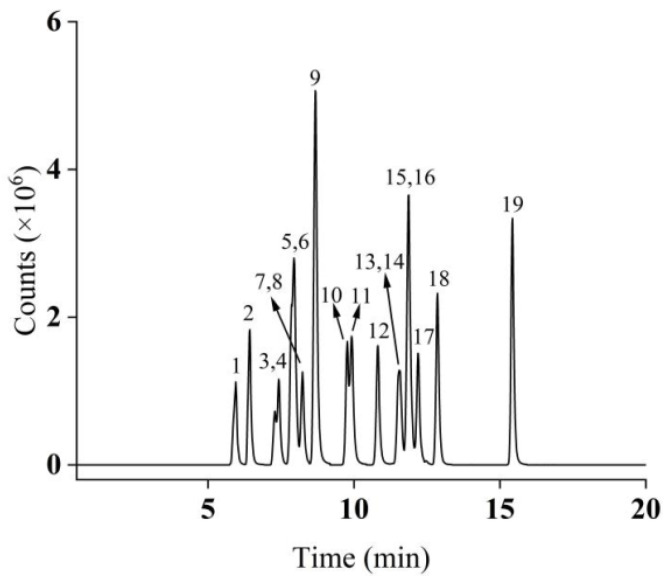
Total ion chromatograms of 19 compounds in MRM mode (peak identification: 1: BPE; 2: BPA; 3: BPB; 4: 4-t-BP; 5: BPAF; 6: BPAP; 7: BPC; 8: 4-BP; 9: BPZ; 10: 4-PP; 11: BPBP; 12: TCBPA; 13: 4-HexylP; 14: BPG; 15: TBBPA; 16: BPP; 17: 4-t-OP; 18: 4-HeptyP; 19: 4-NP).

The optimized MS parameters are detailed in Table 1. Under the optimized chromatographic conditions, the total ion chromatogram (TIC) of the 50 μg/L mixed standard solution is presented in Figure 1.

### 3.4. Optimization of Sample Pretreatment Method

#### 3.4.1. SUPRAS Synthesis and Description

This study developed a streamlined SUPRAS-based LC–MS/MS method for simultaneous quantification of 19 BPs and APs in botanical dietary supplements. Using a spiked capsule, tablet, and oral liquid blanks, we optimized a ternary solvent system for SUPRAS preparation and evaluated its extraction parameters to achieve integrated extraction and cleanup. To develop optimal SUPRAS formulations, the physicochemical interactions between target compounds and solvents were first analyzed. As shown in Table S1, the 19 BPs and APs exhibit medium polar to nonpolar characteristics (LogP range: 3.3–6.8) and contain multiple hydrogen bonding sites. This analysis guided the selection of hydrogen bond-donating SUPRAS components to maximize extraction efficiency. Short-chain alkanols are low-toxicity amphiphiles with tunable size and polarity [41]. SUPRAS, formed spontaneously from alkanol–THF–water ternary mixtures through room-temperature centrifugation, demonstrate remarkable macromolecular exclusion capabilities due to their restricted access properties [25,30].

The SUPRAS protocol was optimized via single-variable analysis to enable single-step extraction/purification of BPs and APs from complex matrices. A fixed total volume (40 mL) was maintained while varying 1-hexanol/THF/water ratios. For parameter screening, 4 mL SUPRAS was used, except during dosage optimization.

#### 3.4.2. Types of Alkyl Alcohols

A series of SUPRAS were synthesized utilizing THF as the dispersant and 1-alkyl alcohols (C5-C10: 1-pentanol to 1-decanol) as amphiphilic components. The extraction performance of these SUPRAS systems was systematically evaluated for eight representative bisphenol and alkylphenol analytes. All experiments were conducted in triplicate (*n* = 3), with recovery rates quantified through spiked blank matrix experiments. As summarized in Table 2, significant differences (*p* < 0.05) were observed in the recoveries of BPE, BPG, and 4-t-OP when different alkanols were used. Given these statistical differences, a compromise optimization was conducted. The 1-hexanol-based SUPRAS system demonstrated superior overall recovery rates relative to systems incorporating longer-chain alkyl alcohols (C7-C10). This enhanced extraction efficiency likely stemmed from the optimal hydrogen-bonding interactions between the C6 alkyl chain and phenolic compounds, as longer chains (≥C7) exhibited progressively weaker hydrogen-bonding capacity with phenolic hydroxyl groups. Based on these findings, the ternary 1-hexanol/THF/water system was selected as the optimal SUPRAS formulation for all subsequent analyses. Within THF/water mixtures, 1-hexanol molecules spontaneously self-assemble to form nanostructured SUPRAS, where the hydroxyl groups organized around aqueous cavities while the hydrocarbon chains remained dispersed in the THF phase [42].

#### 3.4.3. 1-Hexanol Dosage

**Figure 2:**
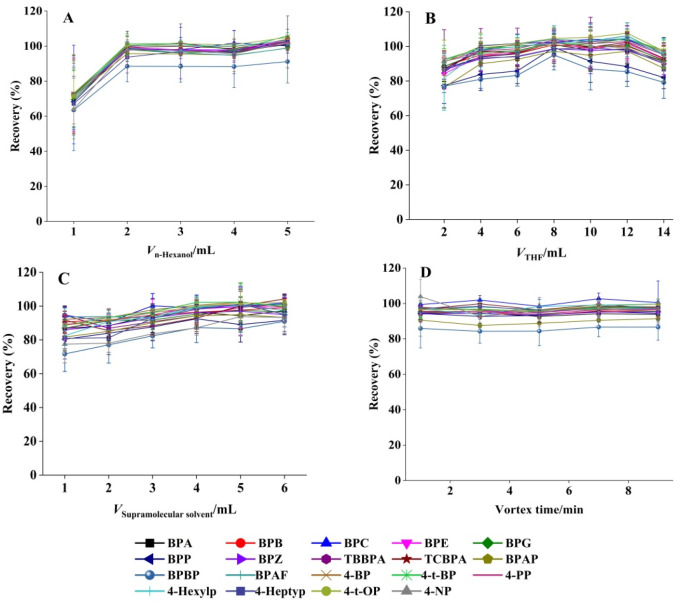
Effect of (**A**) 1-hexanol dosage, (**B**) THF volume, (**C**) SUPRAS volume, and (**D**) vortex duration on the extraction efficiency (mean ± SD, *n* = 3).

The extraction efficiency of 19 target BPs and APs was evaluated as a function of 1-hexanol volume (1.0–5.0 mL). As demonstrated in Figure 2A, analyte recoveries increased proportionally with 1-hexanol volume up to 2.0 mL, reaching a plateau at higher volumes. This saturation effect suggested that 2.0 mL represents the critical threshold for complete SUPRAS assembly, ensuring optimal extraction performance. The concentration of 1-hexanol in SUPRAS is about 0.2 mg/μL, providing sufficient binding sites for BPs and APs. Consequently, this volume was adopted for all subsequent SUPRAS preparations.

#### 3.4.4. THF Volume

THF typically functions as the dispersant in the SUPRAS system. The optimal THF volume was determined by testing a range from 2 to 14 mL while maintaining constant conditions (2 mL 1-hexanol, total volume 40 mL). As shown in Figure 2B, the recovery of all 19 target compounds initially increased with THF volume, reaching maximum efficiency at 8 mL, followed by a gradual decrease at higher volumes. This trend indicated that 8 mL of THF achieved optimal solvent dispersion while maintaining effective phase separation. Accordingly, this volume was selected for all subsequent experiments.

#### 3.4.5. SUPRAS Volume Optimization

Extraction performance was assessed using SUPRAS volumes ranging from 1 to 6 mL. Figure 2C demonstrated that analyte recoveries slightly increased with SUPRAS volume up to 4 mL, reaching a plateau at higher volumes. This volumetric threshold represented the optimal balance between extraction efficiency and solvent economy, and was therefore employed in all subsequent procedures.

#### 3.4.6. Effect of Vortex Duration

Effective vortex mixing is essential for the complete transfer of target analytes from the sample matrix to the SUPRAS phase. We systematically evaluated vortex durations ranging from 1 to 9 min (1, 3, 5, 7, and 9 min; *n* = 3). Figure 2D demonstrates the comparable recovery rates across all tested intervals for the 19 target compounds, confirming rapid analyte partitioning into the SUPRAS phase. Based on these results, a 1 min vortex time was adopted to streamline sample preparation while maintaining quantitative recovery. The enhanced interfacial contact area between target molecules and SUPRAS in this microextraction system promoted faster mass transfer, allowing for rapid extraction completion [43].

The final SUPRAS formulation comprised 2 mL 1-hexanol (5%, *v*/*v*), 8 mL THF (20%, *v*/*v*), and 30 mL H_2_O (75%, *v*/*v*). Following vortex mixing (1 min) and centrifugation (87,012× *g*, 5 min), the supernatant was collected as the ready-to-use SUPRAS phase.

### 3.5. Method Evaluation

#### 3.5.1. Linearity, LODs, and LOQs

The developed method was systematically validated for linearity and sensitivity. However, HPLC–MS/MS analysis identified significant matrix suppression effects for several analytes. Matrix-matched calibration demonstrably reduces matrix interferences compared to solvent-based calibration [44,45]. This partial compensation, combined with its cost-effectiveness and ease of implementation, established matrix-matched calibration as the optimal choice for quantitative analysis in this study. To evaluate method performance, matrix-matched calibration was established using extracts from three representative blank matrices (capsule, tablet, and oral liquid formulations). Among all 19 substances, BPA and 4-PP had a linear range of 1–80 μg/L; the other 17 substances shared a linear range of 5–100 μg/L. As summarized in Table S2, all compounds exhibited satisfactory linear responses within the tested range. The method demonstrated high sensitivity, with limits of detection (LODs) and quantification (LOQs) ranging from 0.0008 to 0.016 μg/g and 0.002 to 0.04 μg/g, respectively, across all three matrices. These results confirmed the method’s reliability for the simultaneous determination of the target analytes in various dosage forms.

#### 3.5.2. Recovery and Precision

The method was validated by spiking blank samples with mixed standards of the 19 target compounds at three concentrations (20, 40, and 80 μg/L) with six replicates per level. The spiked samples were analyzed following the optimized extraction protocol to determine method recovery, relative standard deviation (RSD), and precision. As shown in Table S3, spiking recoveries ranged from 75.0% to 109.5% with RSDs of 0.4–5.7%. As shown in Table S4, intra-day (*n* = 6) and inter-day (*n* = 6) precisions were 0.4–8.1% and 0.7–9.8%, respectively. The validation results confirmed the method’s excellent sensitivity, robust reproducibility, and capability for simultaneous determination of 19 BPs and APs in botanical dietary supplement samples.

#### 3.5.3. Method Comparison with Existing Techniques

A comparison of the developed method with recently reported assays for BPs and APs in foodstuff is shown in Table S5. For simple aqueous matrices, eight BPs and two phenols migrating from food contact materials into food simulants were analyzed by LC–MS [46] with direct injection or simple extraction due to uncomplicated matrices. BPA and two APs in drinking water were processed by vortex-assisted DLLME [23], showing prolonged operation time and suboptimal sensitivity. For complex food samples, sample pretreatment for extraction and cleanup becomes essential. Lucarini et al. [47] detected 16 BPs in canned foods and beverages using a time-consuming QuEChERS procedure. Ultrasound-assisted extraction coupled with SPE was employed for BPA and four analogs in various foods [24], requiring multiple organic solvents with limited analyte coverage. SUPRAS-based extraction was employed to handle 21 BPs and derivatives in solid/liquid samples [48], significantly reducing reagent consumption but requiring nitrogen evaporation/reconstitution steps that extended analysis time. The studies mentioned above were predominantly focused on the detection in simple liquid and food samples, with limited attention given to dietary supplements. For the detection of BPs in dietary and nutrition supplements, Owczarek et al. developed a diethyl ether extraction–GC–MS method [19], which showed longer analysis time and significantly inferior sensitivity (approximately 20~100 fold higher LODs) than our approach. In contrast, this study established a SUPRAS extraction method for the concurrent extraction of 19 BPs and APs from botanical dietary supplements matrices with 4 mL solvent, completing sample preparation within 10 min. The optimized solvent formulation allowed mass production and storage. Large-scale processing was achieved through simple vortexing/centrifugation, streamlining sample preparation, and minimizing solvent usage.

Alternatively, in terms of processing time, number of steps, derivatization requirement, and other aspects, four sample pretreatment methods—liquid–liquid extraction, SPE, QuEChERS, and SUPRAS—were evaluated and compared. As shown in Table 3, the SUPRAS method proved to be more convenient and time efficient. Additionally, the proposed method enabled higher-throughput analysis of both BPs and APs, consumed a well-controlled volume of organic solvent, and offered superior specificity for dietary supplements. The analytical results facilitated health risk assessment of botanical dietary supplement-derived BP and AP exposure.

#### 3.5.4. Sustainability Assessment of the Proposed Method

**Figure 3:**
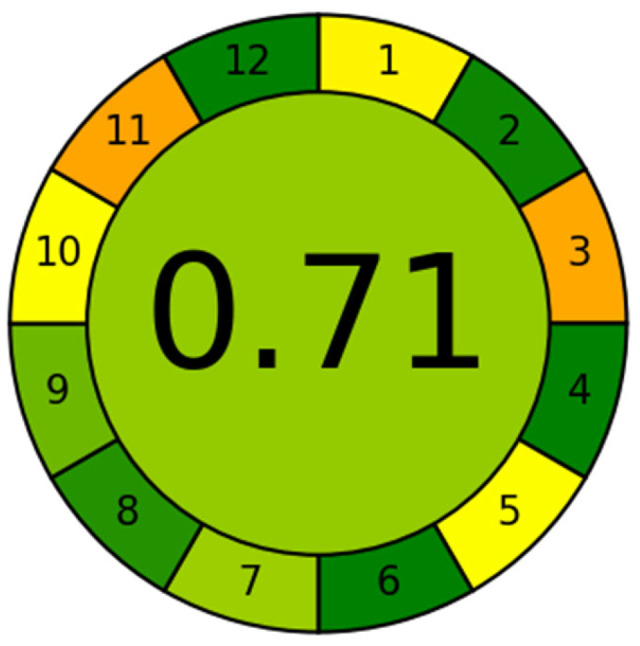
Application of the AGREE approach to evaluate the proposed method. 1. Sample treatment; 2. Sample amount; 3. Device positioning; 4. Sample pretreatment stages; 5. Automation and miniaturization; 6. Derivatization; 7. Waste; 8. Analysis throughout; 9. Energy consumption; 10. Source of reagents; 11. Toxicity; 12. Operator’s safety. In the image, the color of each item transitions from green to yellow and then to orange, indicating that the score obtained for that item decreases progressively.

The sustainability of the proposed SUPRAS-based analytical method was evaluated using the Analytical Greenness Calculator (AGREE), a widely recognized assessment tool [49,50]. AGREE quantitatively scores analytical methods on a scale of 0 to 1 based on the 12 principles of green analytical chemistry, with results visualized as a color-coded pictogram (red–yellow–green scale). A score above 0.6 indicates acceptable method greenness, with higher scores (closer to 1) reflecting superior sustainability [51]. In this study, the AGREE (https://mostwiedzy.pl/wojciech-wojnowski,174235-1/AGREE, accessed on 23 October 2025) evaluation yielded a score of 0.71, as shown in Figure 3, demonstrating that the developed method exhibits good environmental friendliness. The proposed method exhibited notable advantages, including minimal sample requirements, simplified sample processing steps, high analytical throughput, avoidance of derivatization reagents and less toxic chemicals, and enhanced operator’s safety. However, the utilization of LC–MS instrumentation remains essential to achieve the required sensitivity for trace-level detection of BPs and APs. Although currently operated in an offline mode, the method’s sustainability could be further improved by developing online sample pretreatment approaches, adopting greener and safer reagents, and reducing solvent consumption.

### 3.6. BP and AP Determination in Commercial Botanical Dietary Supplements

The developed method was employed to analyze 26 commercial botanical dietary supplement samples. The relevant information, including their codes, dosage forms, packaging materials, and detection results, were presented in Table S6, with the results of the two positive batches separately shown in Table 4. Quantifiable levels of BPA (178.7 μg/kg) and 4-PP (145.3 μg/kg) were identified in a tablet formulation, with BPA (452.6 μg/kg) additionally detected in a capsule product. As EDCs, both BPA (a bisphenol) and 4-PP (an alkylphenol) exhibit estrogenic activity by interacting with estrogen receptors. Current research reveals that botanical dietary supplements may contribute to the ingestion of BPs and APs. It also highlights specific enterprises with serious contamination, calling for targeted surveys to facilitate improvement and for enhanced regulatory oversight to block the market entry of contaminated products. It should be noted that the limited sample size, combined with the nature of the raw plant materials, likely contributed to the low detection rate of APs and BPs in this work.

The detection of BPA and 4-PP in some samples suggests the possibility of contamination through pathways such as (1) plant uptake from polluted environments, with studies confirming BPA presence and metabolism in plants [52] and the ability to penetrate plant cell membranes [53], while BPF was detected in plants used in traditional medicine [37]; and (2) leaching from packaging materials (e.g., bottles, lid linings) into products [54,55]. It is plausible that contamination occurred during production, although this has not been fully substantiated by the available data. Given the limited sample size, future work should expand sampling and trace specific sources by analyzing raw materials, intermediates, finished products, and packaging.

### 3.7. Dietary Exposure and Simplified Risk Assessment

The existing results can serve as a basis for deriving human exposure estimates, which support a simplified preliminary risk assessment. The first consideration involved both the analyte concentration in samples and the daily intake of product. The former is challenged by the limited sample size with a high proportion of non-detect data. Moreover, the authoritative daily intake data of dietary supplements for the population, especially vulnerable groups, were insufficient. On this basis, we established two distinct scenarios for the EDI calculation [39]. In Scenario 1, representing the average exposure, the mean concentration of all samples was used as the content of analytes in the samples. The daily intake was derived from the average of the recommended dosages stated on the product labels. Using worst-case assumptions, the results below the analytical limits were treated as the LOD according to the guideline for left-censored data from the European Food Safety Authority (EFSA) [56]. Thus, the mean concentrations in all samples were 28.0 mg/kg for BPA and 7.1 mg/kg for 4-PP, with an average daily consumption of 2.7 g originated from the data in Tables S6 and S7. The resulting EDIs were 1.3 and 0.3 ng/kg·BW/d for BPA and 4-PP, respectively (Table 5). Scenario 2 models extreme exposure using only the contaminated samples. According to the data in Tables S6 and S8, for sample C11, with a recommended daily intake of 9 g, the EDIs for BPA and 4-PP were calculated to be 26.8 and 21.8 ng/kg·BW/d, respectively. For sample T16 (daily intake: 1.6 g), the EDI for BPA was 12.1 ng/kg·BW/d, while for 4-PP, it was calculated based on the LOD, resulting in an EDI of 0.04 ng/kg·BW/d, as detailed in Table 6.

Because of the unavailability of TDI values for 4-PP, the TDI value of BPA was used for calculating the HQ values for 4-PP. The calculated HQ and HI were as follows: Scenario 1: HQs of 6 (BPA) and 2 (4-PP), giving an HI of 8; Scenario 2: Sample C11 had HQs of 60 and 0.2 (HI = 60), while Sample T16 had HQs of 134 and 109 (HI = 243). An HI value below 1 indicates safety, whereas an HI above 1 implies a potential for adverse health effects in the exposed population [57]. Both the average and extreme exposure scenarios indicated that BPA and 4-PP in botanical dietary supplements pose a potential health risk.

Notably, this study also suffers limitations compared to existing research, with the primary constraint being the small sample size. The absence of dietary intake data in China led to insufficient statistics, supporting only a simplified risk assessment. Nevertheless, this preliminary assessment based on a small sample size provided new clues for risk identification and warrants further investigation. This study also leaves the question of the BPA and 4-PP source unanswered. To address this, future research should investigate whether these low-level pollutants originate from the raw material stage, the processing stage, or the packaging stage.

Considering the non-negligible health risk, continuous attention should be paid to BP and AP exposure via botanical dietary supplements. The method developed here enables future large-scale surveys to define the true contamination profile of these products. EFSA and FAO/WHO [58] set and continually reassess the TDI for BPA. China has banned the use of BPA in the production of infant feeding bottles, lowering the specific migration limit for BPA in food contact materials from 0.6 mg/kg to 0.05 mg/kg, while also introducing a 0.01 mg/L limit in drinking water. However, maximum residue limits for BPA analogs in botanical dietary supplements are yet to be established domestically or by international standards. Furthermore, manufacturers must control production processes to prevent contamination and integrate pollutant monitoring into quality assurance systems. Consumers should recognize that “natural” does not equal “safe” and use such products rationally.

## 4. Conclusions

This study developed a SUPRAS using a 1-hexanol/THF/water ternary system under mild conditions for extracting 12 BPs and 7 APs from commercial botanical dietary supplement samples. After optimizing the SUPRAS preparation, extraction process, and LC–MS/MS conditions, a reliable method for the simultaneous determination of these 19 compounds was established. The validated method demonstrated good reproducibility, satisfactory recoveries, and high sensitivity. Compared with conventional approaches, this method showed simpler operation, higher efficiency, and lower solvent consumption, achieving an AGREE score of 0.71 (out of 1), confirming its environmental friendliness. Application to commercial supplements detected BPA (178.7–452.6 μg/kg) and 4-PP (145.3 μg/kg), indicating potential health risks. Based on the limited sample size, a risk assessment was conducted under two scenarios, providing initial clues regarding the risks of EDCs in botanical dietary supplements.

## Figures and Tables

**Table 1:** The MS/MS parameters for each of 19 compounds.

No.	Compounds	Precursor Ions (*m*/*z*)	Product Ions (*m*/*z*)	DP	CE
1	BPA	227.1	133.0 *, 211.9	−80	−33, −25
2	BPB	241.3	211.3 *, 146.8	−70	−37, −35
3	BPC	255.5	146.8 *, 238.9	−80	−38, −39
4	BPE	213.0	197.3 *, 118.7	−70	−40, −30
5	BPG	311.1	295.1 *, 174.8	−90	−42, −40
6	BPP	345.1	314.9 *, 132.8	−100	−51, −46
7	BPZ	267.0	172.9 *, 222.9	−80	−35, −45
8	TBBPA	542.7	417.4 *, 445.7	−80	−56, −44
9	TCBPA	365.2	313.8 *, 285.8	−80	−37, −44
10	BPAF	335.2	264.2 *, 68.7	−60	−33, −70
11	BPAP	288.7	274.0 *, 210.8	−60	−28, −36
12	BPBP	351.1	273.2 *, 258.0	−80	−35, −34
13	4-BP	149.1	105.7 *	−45	−21
		150.2	106.7	−45	−21
14	4-t-BP	149.2	132.7 *	−60	−25
		150.2	133.6	−60	−23
15	4-PP	163.2	105.7 *	−45	−22
		164.7	106.7	−45	−22
16	4-HexylP	176.8	105.7 *	−50	−23
		178.1	106.7	−50	−23
17	4-HeptyP	191.3	105.7 *	−55	−25
		192.1	106.8	−55	−25
18	4-NP	219.1	105.7 *	−60	−27
		220.1	106.7	−60	−27
19	4-t-OP	205.1	132.7 *	−60	−29
		206.1	133.7	−60	−28

* The quantitative product ion.

**Table 2:** Recoveries (%) of eight representative analytes using SUPRAS with different alkyl alcohols.

Alkyl Alcohols	BPA	BPE	BPG	BPP	4-BP	4-PP	4-HeptyP	4-t-OP
1-Pentanol	95.1 (±5.0) ^a^	94.5 (±5.0)	96.5 (±4.0)	82.0 (±6.1)	107.8 (±3.4)	97.3 (±2.9)	86.8 (±8.6)	94.5 (±3.7)
1-Hexanol	103.5 (±4.1)	103.0 (±5.7)	102.0 (±3.9)	92.9 (±5.5)	96.7 (±2.4)	102.4 (±3.3)	93.0 (±9.6)	100.4 (±2.9)
1-Heptanol	77.0 (±21.3)	91.6 (±5.3)	91.2 (±4.8)	79.5 (±8.6)	92.1 (±4.3)	90.1 (±7.7)	86.3 (±9.6)	90.7 (±2.2)
1-Octanol	93.4 (±9.4)	86.9 (±8.6)	95.2 (±8.7)	86.0 (±13.4)	90.4 (±13.1)	95.7 (±6.5)	89.7 (±12.2)	94.9 (±7.2)
1-Nonanol	90.9 (±0.5)	92.3 (±1.0)	91.7 (±2.2)	92.7 (±12.0)	96.4 (±3.7)	86.8 (±2.0)	84.3 (±8.3)	92.3 (±5.0)
1-Decanol	90.5 (±5.6)	88.4 (±5.0)	73.0 (±7.9)	72.2 (±11.4)	99.2 (±7.9)	93.0 (±10.9)	68.5 (±12.4)	71.1 (±8.5)
*p* value ^b^	0.1283	0.0400	0.0009	0.1572	0.0984	0.1151	0.1362	0.0005

^a^ Data in parentheses indicate the standard deviation from three experimental replicates. ^b^ Calculated by performing an analysis of variance (ANOVA) using R software.

**Table 3:** Comparison of pretreatment methods for bisphenol analysis in dietary supplements and foods.

Comparison Metrics	SUPRAS ^a^	Liquid Extraction [19]	SPE [24]	QuEChERS [47]
Pretreatment time	<10 min	>1.5 h	>1.0 h	>1.5 h
Operation steps	3	5	8	6
Derivatization	No	Yes	No	Yes
Analysis throughout	19	6	5	16
Type of analytes	APs and BPs	BBPs	APs	APs
Organic reagent	4 mL	2 mL	10 mL	10 mL
Method specificity	Dietary supplements	Dietary supplements	Kinds of food samples	Canned foods and beverage

^a^ The present work.

**Table 4:** Levels of BPA and 4-PP found in two botanical dietary supplements samples (μg/kg, *n* = 4). Only positive samples were shown.

Sample No.	Dosage Form	BPA	4-PP
C11	Capsule	452.6 ± 4.6 ^a^	<LOD
T16	Tablet	178.7 ± 5.8	145.3 ± 6.9

^a^ Mean ± SD.

**Table 5:** Assessment of dietary BPA and 4-PP exposure in botanical dietary supplements (Scenario 1).

Compound	Average Concentration (ng/g) ^a^	Daily Consumption ^b^	EDI(ng/kg·BW/d)	TDI (ng/kg·BW/d)	HQ	HI
BPA	28.0	2.7	1.3	0.2	6	8
4-PP	7.1		0.3		2	

^a^ The content of BPA and 4-PP were calculated as the average value of all samples (*n* = 26), with non-detectable data treated as the LOD. ^b^ The daily consumption was calculated based on the average value of the daily dosage indicated on the solid products.

**Table 6:** Assessment of dietary BPA and 4-PP exposure in two positive products (Scenario 2).

Sample	Detected Results (ng/g)		Daily Consumption (g/d)	EDI (ng/kg·BW/d)		TDI (ng/kg·BW/d)	HQ		HI
	BPA	4-PP		EDI_BPA_	EDI_4-PP_		HQ_BPA_	HQ_4-PP_	
C11	452.6	ND	1.6	12.1	0.04 ^a^	0.2	60	0.2	60
T16	178.7	145.3	9.0	26.8	21.8		134	109	243

^a^ EDI_4-PP_ was calculated using the content of 4-PP in the sample as the LOD (1.6 μg/kg).

## Data Availability

The original contributions presented in the study are included in the article/Supplementary Materials; further inquiries can be directed to the corresponding authors.

## Supplementary Materials

The following supporting information can be downloaded at https://www.mdpi.com/article/10.3390/foods14213768/s1, Table S1: Characterization data of the 19 target analytes. Table S2: Linear equation, LODs, and LOQs for 19 analytes in three matrices. Table S3: Recovery and precision data for 19 analytes by SUPRAS–HPLC–MS/MS (n = 6, %). Table S4: The intra-day (n = 6) and inter-day (n = 6) RSDs for 19 compounds in different samples (%). Table S5: Comparison of the proposed method with others reported in the literature. Table S6: Brief sample information and test results. Table S7: Statistical analysis of detection results for BPA and 4-PP in samples. Table S8: Statistics and calculation of daily dosage under two scenarios.
