# Supplementary material for: Supramolecular Solvent-Based Extraction of Bisphenols and Alkylphenols in Botanical Dietary Supplements Prior to HPLC–MS/MS Analysis
Source: Foods. 2025 Nov 3;14(21):3768. doi: 10.3390/foods14213768 (PMC12610146; doi:10.3390/foods14213768)
Supplement: Supplementary file 1 [file foods-14-03768-s001.zip › foods-3919143-supplementary.pdf]

Supplementary Material

Supramolecular Solvent-Based Extraction of Bisphenols and Alkylphenols in Dietary Supplements Prior to HPLC-MS/MS Analysis

Yalei Dong[a], Huijun Liu[c], Yasen Qiao[a,*] and Haiyan Wang[a, *]

[a] National Institute for Food and Drug Control, Beijing 100050, China; dongyalei@nifdc.org.cn (Y.D.)

[b] Department of Food Science and Engineering, Beijing University of Agriculture, Beijing 102206, China; huijunliu78@163.com (H. L.)

Correspondence: 2608753057@qq.com (Y. Q.); Summerwhy163@163.com (H. W.)

Table S1 Characterization data of the 19 target analytes

No.	Types	Compound	CAS	Abbreviation	Chemical formula	Monoisotopic mass (g/mol)	LogP[a]	Hydrogen bond donor count[a]	Hydrogen bond acceptor count[a]
1	Bisphenols	Bisphenol A	80-05-7	BPA	$C_{15}H_{16}O_2$	228.29	3.3	2	2
2		Bisphenol B	77-40-7	BPB	$C_{16}H_{18}O_2$	242.31	3.9	2	2
3		Bisphenol C	79-97-0	BPC	$C_{17}H_{20}O_2$	256.34	4.7	2	2
4		Bisphenol E	2081-08-5	BPE	$C_{14}H_{14}O_2$	214.26	3.9	2	2
5		Bisphenol G	127-54-8	BPG	$C_{21}H_{28}O_2$	312.4	6.3	2	2
6		Bisphenol P	2167-51-3	BPP	$C_{24}H_{26}O_2$	346.5	6.1	2	2
7		Bisphenol Z	843-55-0	BPZ	$C_{18}H_{20}O_2$	268.3	5.4	2	2
8		Tetrabromobisphenol A	79-94-7	TBBPA	$C_{15}H_{12}Br_4O_2$	543.9	6.8	2	2
9		Tetrachlorobisphenol A	79-95-8	TCBPA	$C_{15}H_{12}Cl_4O_2$	366.1	6.5	2	2
10		Bisphenol AF	1478-61-1	BPAF	$C_{15}H_{10}F_6O_2$	336.23	4.5	2	8
11		Bisphenol AP	1571-75-1	BPAP	$C_{20}H_{18}O_2$	290.4	4.4	2	2
12		Bisphenol BP	1844-01-5	BPBP	$C_{25}H_{20}O_2$	352.4	5.6	2	2
13	Alkylphenols	4-Butylphenol	1638-22-8	4-BP	$C_{10}H_{14}O$	150.22	3.4	1	1
14		4-tert-Butylphenol	98-54-4	4-t-BP	$C_{10}H_{14}O$	150.22	3.3	1	1
15		4-Pentylphenol	14938-35-3	4-PP	$C_{11}H_{16}O$	164.24	3.7	1	1
16		4-Hexylphenol	2446-69-7	4-HexylP	$C_{12}H_{18}O$	178.27	4.3	1	1
17		4-Heptylphenol	1987-50-4	4-HeptyP	$C_{13}H_{20}O$	192.3	4.8	1	1
18		4-Nonylphenol	104-40-5	4-NP	$C_{15}H_{24}O$	220.35	5.9	1	1
19		4-tert-Octylphenol	140-66-9	4-t-OP	$C_{14}H_{22}O$	206.32	5.0	1	1

a LogP values, hydrogen bond donor count and hydrogen bond acceptor count obtained from PubChem.

Table S2 Linear equation, LODs, and LOQs for 19 analytes in three matrix

Compound	Linear range (μg/L)	Linear equation			LODs (μg/g)	LOQs (μg/g)
		Slope	Intercept	Relationship coefficient (R)		
BPA	1-80	76101.8 (±3549.7)[a]	-51245.1(±12455.2)	0.9984~0.9993	0.004	0.01
BPB	5-100	27867.1 (±203.2)	6504.8 (±2642.2)	0.9996~0.9998	0.002	0.006
BPC	5-100	4456.1 (±294.4)	-6197.1 (±4313.1)	0.9960~0.9996	0.016	0.04
BPE	5-100	39658.0 (±247.2)	3627.2 (±2862.9)	0.9996~0.9998	0.002	0.006
BPG	5-100	22892.6 (±726.5)	-11190.6 (±1158.5)	0.9996~0.9999	0.002	0.006
BPP	5-100	91015.8 (±11127)	11864.5 (±4608.5)	0.9992~0.9999	0.0016	0.004
BPZ	5-100	152885.0 (±4144.6)	22579.0 (±3606.6)	0.9997~0.9999	0.0008	0.002
TBBPA	5-100	7515.9 (±567.2)	2003.0 (±204.0)	0.9993~0.9998	0.008	0.02
TCBPA	5-100	46329.4 (±1609.5)	-22483.4 (±6114.6)	0.9993~0.9997	0.0008	0.002
BPAP	5-100	81994.3 (±4900.2)	22938.5 (±9032.0)	0.9993~0.9996	0.002	0.006
BPBP	5-100	33123.7 (±1570.5)	1082.2 (±1015.4)	0.9996~0.9999	0.0016	0.004
BPAF	5-100	60992.3 (±278.7)	27183.7 (±8202.0)	0.9996~0.9999	0.0016	0.004
4-BP	5-100	49101.2(±2173.2)	5753.8 (±930.3)	0.9997~0.9999	0.004	0.01
4-t-BP	5-100	36078.4 (±1401.2)	6138.6 (±4368.9)	0.9995~0.9997	0.004	0.01
4-PP	1-80	97316.9 (±7630.7)	60323.5(±18644.8)	0.9982~0.9993	0.0016	0.004
4-HexylP	5-100	43524.3 (±3753.4)	-12471.3 (±1995.7)	0.9992~0.9999	0.0032	0.008
4-HeptyP	5-100	104267.5 (±18982.5)	-1236.9 (±6102.1)	0.9977~0.9999	0.0008	0.002
4-t-OP	5-100	55621.6 (±5018.2)	-51431.0 (±5049.1)	0.9993~0.9999	0.016	0.04
4-NP	5-100	88502.5 (±29702.1)	213918.5 (±17642.9)	0.9937~0.9999	0.0008	0.002

[a] Standard deviations in parentheses were derived from triplicate standard curves across three matrices.

Table S3 Recovery and precision data for 19 analytes by SUPRAS-HPLC-MS/MS (n = 6, %)

Compound	Added (μg/L)	Capsule		Tablet		Oral liquid	
		Recoveries	RSD	Recoveries	RSD	Recoveries	RSD
BPA	20.4	93.9	2.6	92.4	1.3	93.5	0.7
	40.8	84.3	1.4	87.0	0.9	108.0	0.8
	81.6	93.4	0.6	89.4	0.6	101.8	0.8
BPB	20.3	91.8	3.6	95.2	1.3	95.4	0.8
	40.6	83.3	1.2	88.3	1.5	108.4	1.4
	81.1	93.7	0.4	90.2	1.6	101.5	1.6
BPC	20.1	84.8	4.8	94.5	2.1	91.6	3.3
	40.1	94.1	2.7	91.9	3.6	109.5	2.4
	80.2	99.0	2.7	103.6	3.9	109.2	2.9
BPE	20.5	92.2	2.3	88.3	1.3	85.4	0.8
	41.0	96.0	2.5	88.1	1.4	107.9	0.8
	81.9	90.3	0.9	89.9	0.4	101.9	1.0
BPG	20.0	85.4	2.5	89.4	3.2	86.2	1.3
	40.1	98.1	2.0	90.3	1.4	104.5	1.3
	80.1	88.9	1.8	91.4	1.0	99.6	1.0
BPP	20.0	88.8	2.2	86.4	3.8	81.2	1.0
	40.0	99.1	3.5	80.3	3.8	102.4	1.2
	79.9	90.4	1.1	79.1	2.4	96.4	1.0
BPZ	20.1	91.7	3.0	91.8	2.4	87.1	0.7
	40.2	93.0	2.1	87.2	1.3	106.4	1.7
	80.4	89.9	0.7	89.3	0.7	100.2	1.5
TBBPA	19.9	85.2	2.9	84.7	2.3	91.8	1.3
	39.9	92.2	1.8	80.5	3.3	97.0	1.5
	79.8	87.9	1.1	78.2	3.3	89.1	1.6
TCBPA	19.6	81.2	3.2	86.5	1.4	93.2	1.7
	39.3	85.9	3.1	84.8	1.0	102.7	1.7
	78.6	86.9	1.2	90.6	1.0	99.1	0.8
BPAP	20.1	87.8	2.4	83.9	2.8	81.3	2.1
	40.2	88.0	2.8	78.2	3.0	102.4	1.0
	80.4	85.8	1.6	76.1	0.9	95.4	2.0
BPBP	19.8	88.3	1.9	85.9	3.1	75.0	2.2
	39.5	87.5	1.2	81.1	1.9	101.5	2.5
	79.0	86.6	1.3	84.0	1.4	95.5	1.4
BPAF	20.7	89.1	3.8	91.9	1.0	95.5	0.7
	41.4	87.7	0.9	87.1	2.9	108.1	1.1
	82.9	89.0	1.4	92.0	0.7	103.1	1.1

	20.1	91.3	1.4	88.3	1.1	83.0	1.6
4-BP	40.2	92.0	1.3	85.7	0.9	105.4	0.9
	80.3	86.6	1.2	86.5	0.8	98.3	1.0
	20.0	92.2	1.5	89.3	2.0	81.1	2.7
4-t-BP	40.1	90.9	2.1	89.7	1.2	107.2	1.3
	80.2	89.6	0.7	89.8	1.1	100.4	1.6
	19.7	93.4	1.4	91.3	1.5	86.0	1.3
4-PP	39.3	92.8	2.6	87.3	1.8	106.4	0.9
	78.6	87.2	0.9	88.9	1.0	98.7	1.6
	20.1	92.6	1.5	83.4	2.5	77.9	1.6
4-HexylP	40.3	90.0	1.8	81.5	3.1	105.2	1.0
	80.6	87.1	0.8	82.6	1.0	97.5	0.5
	19.6	102.7	5.7	97.5	3.6	94.0	0.7
4-HeptyP	39.2	90.6	1.5	100.9	2.6	104.4	1.1
	78.5	94.6	2.5	105.4	1.1	98.6	1.2
	20.5	86.7	2.4	83.7	1.7	79.0	0.9
4-t-OP	40.9	89.3	3.7	86.2	1.2	100.6	2.0
	81.8	87.1	0.8	84.0	1.3	93.0	0.5
	20.3	87.7	2.1	92.5	1.1	89.5	0.6
4-NP	40.5	90.0	2.5	84.4	0.8	104.6	2.2
	81.0	85.3	3.1	106.1	1.3	98.6	2.4

Table S4 The intra-day (n=6) and inter-day (n=6) RSDs for 19 compounds in different samples (%)

Compound	Capsule		Tablet		Oral liquid	
	inner-day	inter-day	inner-day	inter-day	inner-day	inter-day
BPA	2.1	6.5	0.8	1.4	0.8	3.3
BPB	1.4	6.4	1.4	1.6	0.4	3.3
BPC	4.5	6.7	3.6	3.9	1.1	9.7
BPE	2.8	9.8	1.9	1.9	0.8	2.1
BPG	4.8	6.9	3.0	3.4	1.2	4.2
BPP	5.0	7.9	3.9	5.0	4.0	5.3
BPZ	2.6	8.6	2.3	2.1	1.6	3.5
TBBPA	2.9	6.2	1.1	3.2	2.2	4.0
TCBPA	2.2	5.6	2.5	3.5	0.9	4.9
BPAP	2.8	9.6	1.9	2.5	1.9	2.0
BPBP	3.9	8.0	1.6	1.9	2.0	3.2
BPAF	2.2	7.5	2.5	3.4	1.6	5.9
4-BP	3.6	9.8	1.6	1.9	1.2	1.1

4-t-BP	4.7	8.9	0.5	2.2	1.1	2.4
4-PP	2.6	9.2	1.5	2.8	1.7	0.7
4-HexylP	3.9	9.2	0.6	3.3	2.1	3.3
4-HeptyP	2.9	7.5	1.3	2.0	1.3	2.9
4-t-OP	8.1	8.6	3.2	4.4	0.8	1.4
4-NP	2.0	7.4	4.9	5.3	3.8	5.0

Table S5 Comparison of the proposed method with others reported in literatures

Sample matrix	Sample type	Analytes	Detection method	Pretreatment method	Extraction solvent	Pretreatment time	LOD	Reference
Simple aqueous matrices	Food simulants from food contact materials	8 bisphenols and 2 phenols	ASAP-LC-MS/MS	Direct injection or extract	25 mL acetonitrile	not provided	0.53~29.6 μg/L	[1]
	Drink water	BPA and 2 APs	HPLC-DAD	Vortex-assisted binary solvent dispersive liquid-liquid microextraction	3 mL acetone	> 2 h	80~210 ng/L	[2]
Food matrices	Canned foods and beverage	16 bisphenols	GC-MS	QuEChERS	10 mL acetonitrile	> 1.5 h	40~1660 ng/L	[3]
	Vegetables, dairy products, seafood products, condiments, Beverages, oils & fats and others)	BPA and 4 analogues	LC-MS/MS	Ultrasonic extraction and SPE	5 mL ethyl acetate, 8 mL methanol, 6 mL acetonitrile	> 0.5 h	0.003~0.015 ng/g	[4]
	Food, dust and biological fluids	21 bisohenols and derivatives	LC-MS/MS	SUPRAS extraction with nitrogen dry	0.2 mL or 0.5 mL solvent	> 0.5 h	0.06~0.81 ng/g	[5]
Dietary supplements	Dietary and nutritional supplements	6 bisphenols	GC-MS	Extracted with diethyl ether and derivatization	1 mL water, 2 mL diethyl ether	> 1.5 h	90~310 ng/g	[6]
	Botanical dietary supplements	19 bisphenols and alkylphenols	LC-MS/MS	Directly extract with SUPRAS	4 mL of SUPRAS	< 10 min	0.8~16 ng/g	The present method

Table S6 Brief Sample Information and Test Results

Sample No.	Dosage form	Packaging materials	Recommended daily dosage[a]	Detection results[b] (μg/kg)	
				BPA	4-PP
C1	Capsule	HDPE	1.2 g	<LOD	<LOD
C2		Aluminum-Plastic	2.7 g	<LOD	<LOD
C3		HDPE	2.7 g	<LOD	<LOD
C4		PET	1.6 g	<LOD	<LOD
C5		HDPE	2.1 g	<LOD	<LOD
C6		HDPE	2.7 g	<LOD	<LOD
C7		PS	3.0 g	<LOD	<LOD
C8		Aluminum-Plastic	0.6 g	<LOD	<LOD
C9		PET	2.1 g	<LOD	<LOD
C10		PP	1.8 g	<LOD	<LOD
C11		HDPE	1.6 g	452.6±4.6	<LOD
C12		HDPE	2.4 g	<LOD	<LOD
T13	Tablet	HDPE	1.8 g	<LOD	<LOD
T14		HDPE	2.0 g	<LOD	<LOD
T15		Aluminum-Plastic	3.6 g	<LOD	<LOD
T16		HDPE	9.0 g	178.7±5.8	145.3±6.9
T17		Aluminum-Plastic	4.8 g	<LOD	<LOD
L18	Oral liquid	Glass	30 mL	<LOD	<LOD
L19		Glass	50 mL	<LOD	<LOD
L20		Glass	60 mL	<LOD	<LOD
L21		Glass	20 mL	<LOD	<LOD
L22		Glass	30 mL	<LOD	<LOD
L23		Glass	20 mL	<LOD	<LOD
L24		Glass	30 mL	<LOD	<LOD
L25		Glass	40 mL	<LOD	<LOD
L26		Glass	50 mL	<LOD	<LOD

HDPE: High-Density Polyethylene; PET: Polyethylene Terephthalate; PS: Polystyrene; PP: Polypropylene.

[a] The information was collected directly from the product's outer packaging.

[b] Only the detection results for BPA and 4-PP were presented, with the remaining 17 compounds all below the LOD.

Table S7 Statistical analysis of detection results for BPA and 4-PP in samples

Compound	Detection Frequency (%)	Range (μg/kg)	Detected Amount (μg/kg)		95th percentile
			Mean ± SD[a]	Mean ± SD[b]	
BPA	7.7 (2/26)	ND-452.6	28.0±93.1	24.3±94.1	121.5
4-PP	3.8 (1/26)	ND-178.7	7.1±28.2	5.6±28.5	1.6

[a] All results below the LOD were calculated as the LOD.

[b] All results below the LOD were calculated as 0.

Table S8 Statistics and calculation of daily dosage under two scenarios

Category		Sample No.	Sample Count	Daily dosage[c] (g/d)	Average daily dosage (g/d)
All samples	Scenario I[a]	C1-T17	17	0.6-9	2.7
Positive samples	Scenario II[b]	C11	1	1.6	-[d]
		T16	1	9.0	

[a] Scenario I represents the average exposure

[b] Scenario II represents the extreme exposure

[c] The daily dosage adopted here is that recommended by the manufacturer as indicated on the product outer packaging.

[d] Not required

References

[1] M.J. Dueñas-Mas, C. de Dios-Pérez, A. Ballesteros-Gómez, S. Rubio. Supramolecular solvent extraction and ambient mass spectrometry for the determination of organic contaminants in food packaging material. Chemosphere, 324 (2023), 138359. https://doi.org/10.1016/j.chemosphere.2023.138359

[2] M. A. Vargas-Muñoz, D.L. Montoya-Cárdenas, L. Bonilla, E. Palacio. Vortex-assisted binary solvent dispersive liquid–liquid microextraction for the determination of two 4-alkylphenols and Bisphenol A in drinking water by HPLC-DAD. Microchem. J, 215 (2025), 114167. https://doi.org/10.1016/j.microc.2025.114167

[3] F. Lucarini, R. Gasco, D. Staedler. Simultaneous Quantification of 16 Bisphenol Analogues in Food Matrices. Toxics, 11 (2023), 665. https://doi.org/10.3390/toxics11080665

[4] H. Shaaban, A. Mostafa, A.M. Alqarni, Y. Almohamed, D. Abualrahi, D. Hussein, M. Alghamdi. Simultaneous determination of bisphenol A and its analogues in foodstuff using UPLC-MS/MS and assessment of their health risk in adult population. J. Food Compos. Anal., 110 (2022), 104549. https://doi.org/10.1016/j.jfca.2022.104549

[5] N. Caballero-Casero, S. Rubio. Comprehensive supramolecular solvent-based sample treatment platform for evaluation of combined exposure to mixtures of bisphenols and derivatives by liquid chromatography-tandem mass spectrometry. Anal. Chim. Acta, 1144 (2021), 14. https://doi.org/10.1016/j.aca.2020.11.057

[6] K. Owczarek, E. Waraksa, E. Kłodzińska, Y. Zrobok, M. Ozimek, D. Rachoń, B. Kudłak, A. Wasik, Z. Mazerska. Validated GC–MS method for determination of bisphenol a and its five analogues in dietary and nutritional supplements. Microchem. J, 180 (2022), 107643. https://doi.org/10.1016/j.microc.2022.107643
